# Processing, Performance Properties, and Storage Stability of Ground Tire Rubber Modified by Dicumyl Peroxide and Ethylene-Vinyl Acetate Copolymers

**DOI:** 10.3390/polym13224014

**Published:** 2021-11-20

**Authors:** Paulina Wiśniewska, Łukasz Zedler, Krzysztof Formela

**Affiliations:** Department of Polymer Technology, Faculty of Chemistry, Gdańsk University of Technology, Gabriela Narutowicza 11/12, 80–233 Gdańsk, Poland; paulina.wisniewska1@pg.edu.pl (P.W.); lukasz.zedler@pg.edu.pl (Ł.Z.)

**Keywords:** ground tire rubber, recycling, modification, ethylene-vinyl acetate copolymers, extrusion, performance properties

## Abstract

In this paper, ground tire rubber was modified with dicumyl peroxide and a variable content (in the range of 0–15 phr) of ethylene-vinyl acetate copolymers characterized by different vinyl acetate contents (in the range of 18–39 wt.%). Modification of ground tire rubber was performed via an auto-thermal extrusion process in which heat was generated during internal shearing of the material inside the extruder barrel. The processing, performance properties, and storage stability of modified reclaimed ground tire rubber were evaluated based on specific mechanical energy, infrared camera images, an oscillating disc rheometer, tensile tests, equilibrium swelling, gas chromatography combined with a flame ionization detector, and gas chromatography with mass spectrometry. It was found that the developed formulas of modified GTR allowed the preparation of materials characterized by tensile strengths in the range of 2.6–9.3 MPa and elongation at break in the range of 78–225%. Moreover, the prepared materials showed good storage stability for at least three months and satisfied processability with commercial rubbers (natural rubber, styrene-butadiene rubber).

## 1. Introduction

According to ASTM D 1566, entitled “Standard terminology relating to rubber”, the term reclaimed rubber is defined as “vulcanized rubber that has been thermally, mechanically, and/or chemically plasticized for use as a rubber diluent, extender, or processing aid”. Standard ASTM D1566 does not define devulcanization or devulcanized rubber. The term devulcanization is defined in the ASTM D 6814 standard, entitled “Standard test method for determination of percent devulcanization of crumb rubber based on crosslink density”. According to this definition, devulcanization is the process of breaking down chemical cross-links in cured rubber.

Different methods of rubber devulcanization (selective scission of cross-links) and reclaiming (scission of cross-links and the main chain degradatio) are comprehensively described in several works [1,2,3].

However, it should be noticed that both mentioned processes allow the conversion of cross-linked rubber into so-called reclaimed rubber, a material suitable for further processing, formulation, and vulcanization [4]. The reclaimed rubber structure is formed by a gel fraction and sol fraction, which indicates that reclaimed rubber can be considered a multicomponent, heterophase composite [5].

The gel fraction is a cross-linked rubber phase (usually filled with carbon black as a reinforcement), which remains after the devulcanization/reclaiming process. The content and size of this phase decrease with a higher reclaiming degree. On the other hand, the sol fraction phase content in reclaimed rubber is a combination of the devulcanized, degraded, or plasticized rubber phase. The higher degree of main chain scission resulted in a decrease in the molecular weight of the polymer and an increase of polydispersity, which usually have a negative effect on the mechanical properties of reclaimed rubber.

In fact, the main difference between rubber reclaiming and devulcanization is based on the subjective assessment related to the analysis of the main chain degradation degree, which is usually supported by using Horikx theory [6,7]. However, it should be mentioned that equilibrium swelling conditions (e.g., initial sample weight, extraction temperature and time, solvent type, etc.) might affect the parameters used in the Horikx theory for the determination of the main chain degradation level [8].

It is well described in the literature that conditions of ground tire rubber (GTR) reclaiming/devulcanization and/or modification affect the processing and performance properties of reclaimed rubbers [9,10,11] and, as a consequence, the interfacial interactions between the matrix and reclaimed rubbers [12,13,14].

Saiwari et al. [15] indicated that for efficient devulcanization of tire rubbers (styrene-butadiene rubber, butadiene rubber, natural rubber, chlorinated butyl rubber) the temperature of the process should be kept as low as possible. Shi et al. [16] studies showed that the recommended GTR reclaiming method should be performed in an oxygen-free atmosphere, without high shear force and at a relatively low temperature.

Research trends showed that low-temperature devulcanization is gaining more and more attention [17,18,19]. The main advantages of low-temperature devulcanization are related to limited main chain scission (the devulcanization mechanism is preferable) and also to significantly reduced levels of volatile organic compounds formulated during rubber reclaiming [20]. Moreover, using low-temperature devulcanization might support the cross-linked rubber self-heating phenomenon [21] related to internal friction of the material, which affects energy consumption of the process [22] and, as a consequence, the final price of the reclaimed rubber.

On the other hand, the processing of cross-linked rubbers at low-temperature devulcanization might cause technological problems, which can be partially resolved, e.g., by using high-torque extruders, reduced throughput, or optimization of screw configuration [23].

Another interesting approach to resolving this issue is the application of thermoplastics, which might act like plasticizers or binders during GTR reclaiming [24,25]. In this field, the most popular additive is a trans-polyoctenamer (tradename: Vestenamer^®^ 8012) that is commercially dedicated to rubber recycling [26].

Recent works showed [27,28] that the application of relatively small amounts (10–25 wt.%) of common and easily available thermoplastics (e.g., low-density polyethylene, polypropylene, ethylene-vinyl copolymers) might enhance the thermal stabilization of extrusion and allow the formulation of reclaimed rubber characterized by a higher sol fraction and/or performance properties. However, in the mentioned research works, the processing temperature during rubber devulcanization was very high (in the range of 210–270 °C). Thermogravimetric analysis of GTR showed that at ~250–260 °C, a loss of 2% of the weight of the sample might be observed (measurements performed in nitrogen and air) [29], which is related to the evaporation of low-molecular compounds (e.g., plasticizers) present in GTR and also partial decomposition of cross-linked rubber. These estimations showed that for GTR devulcanization trials in extruders with a laboratory throughput of 1 kg/h, it might result in the emission of volatile organic compounds (VOCs) at the level of ~20 g/h (this estimation does not include emission during the cooling of extruded material). The information about VOCs’ emission profiles during processing and use of prepared materials is crucial for further up-scaling and commercialization-related procedures.

Our previous work [30] showed that using of a relatively small amount (10 phr) of ethylene-vinyl acetate copolymer during low-temperature devulcanization resulted in the improvement of the tensile properties of the modified reclaimed GTR, which were also higher compared to GTR modified by trans-polyoctenamer (an additive commonly used in waste rubber recycling). Furthermore, the results showed that using of thermoplastics in GTR limits the emission of volatile organic compounds.

However, as presented above, research works regarding the application of thermoplastics as modifiers in low-temperature devulcanization of cross-linked rubber are rather limited. To the best of our knowledge, there is no published information about the processing, performance properties, and storage stability of ground tire rubber modified by dicumyl peroxide and ethylene-vinyl acetate copolymers, which was the subject of our investigation.

In this work, GTR modification by dicumyl peroxide and ethylene-vinyl acetate copolymers was performed via auto-thermal extrusion. The effects of ethylene-vinyl acetate copolymer content (0–15 phr) and thermoplastic modifier structure (vinyl acetate content: 18–39 wt.%) on the processing and performance properties of the obtained modified GTR were investigated by measurement of energy consumption, temperature of GTR after treatment, curing behavior, tensile properties, equilibrium swelling, and volatile organic compounds emission profiles. Moreover, the storage stability of the prepared materials and blends of commercial rubbers (natural rubber, styrene-butadiene rubber) with modified GTR (in a ratio of 75/25; 50/50 and 25/75 wt.%) were also studied.

## 2. Materials and Methods

### 2.1. Materials

In order to produce the studied materials, the following components were used:

Ground tire rubber (GTR) obtained from passenger and truck tires, with particle sizes up to 0.6 mm, was received from Grupa Recykl S.A. (Śrem, Poland). The basic components of GTR are natural rubber (NR), styrene-butadiene rubber (SBR), butadiene rubber (BR), additives (curing system, activators, plasticizers, etc.), carbon black, silica, and ash [29].

Escorene Ultra™ FL00218, Escorene Ultra™ UL04533EH2, and Escorene Ultra™ UL05540EH2 are copolymers of ethylene and vinyl acetate (EVA) characterized by different vinyl acetate (VA) content in the range of 18–39 wt.% EVA copolymers were supplied by ExxonMobil Chemical (Machelen, Belgium). For clarity, in this study, EVA copolymers were coded as EVA-18%, EVA-33%, and EVA-39%, respectively. A summary with the characteristics of the used copolymers is presented in Table 1.

Dicumyl peroxide (DCP) is a radical initiator, commercially used in the vulcanization of rubber, cross-linking, and synthesis of copolymer blends, with a molar mass of 270.37 g/mol, a density of 1.56 g/cm^3^, a melting temperature of 39–41 °C, and a half-life temperature (at 0.1 h) of 162 °C. The peroxide was supplied by Pergan GmbH (Bocholt, Germany).

KER^®^ 1502 styrene-butadiene rubber (SBR), a standard grade of styrene-butadiene rubber, was supplied by Synthos S.A (Oświęcim, Poland). It is obtained by low-temperature emulsion polymerization based on a mixture of fatty acid and resin soaps. It contains approximately 22–25% chemically bound styrene.

Natural rubber (NR), type RSS with a density of 0.92 g/cm^3^, was kindly supplied by Guma-Pomorska (Głobino, Poland).

### 2.2. Sample Preparation

#### 2.2.1. GTR Modification and Curing

Reclaimed GTR modified by thermoplastics was prepared using a co-rotating twin-screw extruder model EHP 2 × 20 Sline from Zamak Mercator Sp. z o.o. (Skawina, Poland) with an L/d ratio of 40. The screw’s diameter was 20 mm, and the rotational speed was equal to 150 rpm. The extruder was equipped with eleven heating zones. The temperatures in the individual heating zones (from hopper to extrusion die) on the barrel of the extruder were 60/85/105/115/110/115/130/130/120/120 °C. After process stabilization, the heaters were turned off to provide auto-thermal conditions. Prior to processing, GTR was premixed with dicumyl peroxide in a constant ratio of 100:2 (GTR/DCP). All components, the GTR/DCP premix, and 0–15 phr of the thermoplastics (Escorene Ultra EVA FL00218, 04533EH2, UL05540EH2) were directly introduced into a hopper with a throughput in the range of 5.0–5.75 kg/h (0–15 phr of modifier). The feeding system was provided by Hydrapress Sp. z o.o. (Białe Błota, Poland). For each studied composition, samples were collected for at least 30 min. After extrusion, the material was cooled and formulated into thick sheets using a two-roll mill.

The obtained modified GTR was formed into the shape of 2 mm thick plates by pressing under a pressure of 10 MPa using an electric heated hydraulic press PH-90 manufactured by ZUP Nysa (Nysa, Poland). During compression, approximately 60 g of modified GTR was used to make one plate. All modified GTR was pressed at 170 °C according to the previously determined optimal cure time.

#### 2.2.2. Preparation of Rubber/Modified GTR Blends

Rubber/modified GTR blends were prepared using a two-roll mill model 14201/P2 from Buzuluk (Komalov, Czech Republic). For blend preparation, two types of rubbers commonly used in tire manufacturing were used: styrene-butadiene rubber (SBR) and natural rubber (NR). The rubber/modified GTR blends (without an additional curing system) were prepared in ratios of 75/25; 50/50; 25/75 wt.% The following two-roll mill settings were used: ambient temperature, friction equaling 1.08, and a gap width varying between 0.2 and 3 mm. Rubber/modified GTR blends were vulcanized at temperatures of 180 °C and pressures of 4.9 MPa for the determined optimal vulcanization time.

### 2.3. Measurements

During the modification of GTR, the energy consumption was determined by two methods. The first was based on the direct measurement of energy consumption using an electrical energy meter installed in the line. This analyzed the consumption of all extruder components; however, the main source of energy consumption was related to the barrel heating and the drive motor. The second method determined the specific mechanical energy (*SME*), expressed in kWh/kg, and can be calculated using Equation (1):

(1) $$SME=\frac{N}{Q}$$

where *N* is the consumption of drive motor power (kW), and *Q* is a throughput (kg/h).

The temperature distribution of mGTR was measured using infrared thermal imagining camera model Testo 872 (Testo SE & Co. KGaA, Lenzkirch, Germany), directly from the die of the extruder.

The Mooney viscosity of the rubber compounds was measured at 100 °C using a Mooney viscosimeter MV2000 (Alpha Technologies, Hudson, OH, USA) according to ISO 289-1.

Curing characteristics were investigated according to ISO 3417, using a Monsanto R100S rheometer with an oscillating rotor (Monsanto Company, St. Louis, MO, USA). The cure rate index (*CRI*) was calculated according to Equation (2):

(2) $$CRI=\frac{100}{t_{90}-t_{1}}$$

where *t*_90_—optimum vulcanization time, min; *t*_1_—scorch time, min.

The aging resistance at elevated temperatures was determined using an R_300_ parameter [31], which is the percentage reversion degree after a period of 300 s calculated from the time of reaching the maximum torque value. R_300_ was calculated according to Formula (3):

(3) $$R_{300}=\frac{M_{H}-M_{300s}}{M_{H}}\times 100\%$$

where *M_H_*—maximum torque, dNm; *M*_300s_—torque 300 s after maximum torque, dNm.

Tensile strength and elongation at break of the obtained samples were tested according to the standard ISO 37 using a Zwick Z020 testing machine (Ulm, Germany) with a load capacity of 20 kN. Tensile tests were performed at a cross-head speed of 500 mm/min. Direct extension measurements were conducted periodically using an extensometer with sensor arms. Hardness was determined using a Zwick 3130 durometer Shore A (Ulm, Germany) in accordance with the standard ISO 7619-1. The reported results of the tensile tests and hardness are the means of at least five measurements per sample.

Abrasion resistance was measured by using an abrasion tester from Gibitre Instruments (Italy) according to the standard ISO 4649. The abrasion resistance value is the average of at least three measurements per sample.

The density of the samples was measured by the Archimedes method in accordance with ISO 2781 while using an analytical balance model AS 110.R2 from Radwag (Radom, Poland). The test was carried out at room temperature, and it consisted of weighing the material in air and then in a methanol medium. The density value is the average of at least three measurements per sample.

The swelling degree of the blends (approx. 0.2 g samples) as a function of time was determined by equilibrium swelling in toluene (at room temperature). The swelling degree was calculated according to Formula (4):

(4) $$Q=\frac{m_{t}-m_{0}}{m_{0}}\times 100\%$$

where *Q*—swelling degree, %; *m_t_*—a mass of the sample swollen after time t, g; *m*_0_—an initial mass of the sample, g.

The sol fraction was determined on the basis of the mass difference of the initial sample and the dried sample after extraction according to Equation (5). The remaining part is a gel fraction (6):

(5) $$F_{sol}=\frac{m_{0}-m_{k}}{m_{0}}\times 100\%$$

(6) $$F_{gel}=100\%-F_{sol}$$

where *F_sol_*—the content of sol fraction, %; *F_gel_*—the content of gel fraction, %; *m*_0_—an initial mass of the sample, g; and *m_k_*—a mass of the dried sample after extraction, g.

Cross-link density was determined according to the Flory–Rehner Equation (7) [32]:

(7) $$V_{e}=\frac{-\left[\ln\left(1-V_{r}\right)+V_{r}+\chi V_{r}^{2}\right]}{\left[V_{1}\left(V_{r}^{\frac{1}{3}}-\frac{V_{r}}{2}\right)\right]}$$

The Flory–Rehner equation is correct for non-filled compounds. The presence of rubber waste in the examined materials causes them to have a high content of carbon black. Therefore, the Kraus correction should be included in order to calculate the actual cross-link density [33]. The correction was calculated in accordance with Equations (8) and (9):

(8) $$\nu_{after\;correction}=\frac{\nu_{e}}{1+K\times \Phi }$$

(9) $$\Phi =\frac{\phi_{f}\times \rho_{r}\times m_{0}}{\rho_{f}\times m_{dry}}$$

where *ν_e_*—the measured chemical cross-link density, mol/cm^3^; *ν*_*after correction*_—the actual chemical cross-link density, mol/cm^3^; *K*—constant characteristic of the filler but independent of the solvent; *ϕ_f_*—the volume fraction of filler in the sample which is calculated; *ρ_r_*—the density of studied compound, g/cm^3^; *m*_0_—the weight of the sample before extraction, g; *ρ_f_*—the density of filler, g/cm^3^; *m_dry_*—the weight of the sample after extraction, g.

A sampling of volatile organic compounds (VOCs) emitted to the atmosphere during the reactive extrusion of the modified GTR was performed using a passive sampling technique with a Radiello^®^ diffusing sampling system (Fondazione Salvatore Maugeri, Padova, Italy). Radiello^®^ diffusive passive samplers are based on three main components: a microporous polyethylene diffusion membrane, a plastic tripod dedicated for installation of the sampler, and a cylindrical steel net filled with graphitized charcoal Carbograph 4 as a sorption bed. A sampling of VOCs directly from the extrusion die was performed for 30 min via the Radiello^®^ system. VOCs collected on Radiello^®^ diffusive passive samplers were liberated using a thermal desorption technique (Unity v.2, Markes International Ltd., Pontyclun, UK), connected with gas chromatography (Agilent Technologies 6890) combined with a mass spectrometer (5873 Network Mass Selective Detector, Agilent Technologies). More detailed information about this methodology is presented in the works [34,35,36].

The level of total volatile organic compounds’ (TVOCs) emissions from uncured and cured modified GTR was determined using microscale stationary emission chamber—Markes’ Micro-Chamber/Thermal Extractor™—μ-CTE™ 250 (Markes International Ltd., Llantrisant, UK) in which samples of approx. 2.0 g were conditioned for 20 min at 40 °C. Volatile organic compounds emitted from the modified GTR were collected using stainless steel tubes filled with Tanax TA as a sorption bed (Markes International Ltd., Llantrisant, UK) under the influence of a carrier gas (nitrogen) with a rate of 25 mL/min. Then, the adsorbed analytes were released through a two-stage process of thermal desorption (Markes Series 2 Thermal Desorption Systems; UNITY/TD100, Llantrisant, UK). Quantitative analysis of TVOCs released from modified GTR was performed on a GC-FID system (Agilent 7820A GC, Agilent Technologies, Inc., Santa Clara, California, USA). More detailed information about the equipment and methodology used is presented in the works [37,38,39].

Acetophenone (a by-product of dicumyl peroxide decomposition) concentration was determined using a static headspace and gas chromatography-mass spectrometry (SHS-GC-MS). All experiments were conducted using a Shimadzu GC2010 PLUS GC-MS equipped with a split/splitless inlet. The GC-MS system was equipped with an AOC5000 Headspace Auto-Sampler. During analysis, a vial with 0.5 g of the sample and also with 10 μL of standard (toluene in methanol, concentration: 1000 ppm) was transported by the injection unit from the tray to the agitator. The headspace sample was incubated at 100 °C for 20 min. When the sample reached equilibrium, the headspace sample was drawn from the vial (syringe temp. 110 °C) and injected into the GC injector.

The morphology of the surface created by breaking the samples in the tensile test at the speed of 500 mm/min was observed with a JEOL 5610 scanning electron microscope (SEM) (Tokyo, Japan). Before measurement, the samples were covered with a fine gold layer in order to increase their conductivity in a vacuum chamber.

To highlight the concept of the research, the sample preparation protocol and methodology for each group of materials studied in this work, i.e., modified GTR and rubber/modified GTR blends, are summarized in Table 2.

## 3. Results and Discussion

### 3.1. GTR Modification

**Figure 1:**
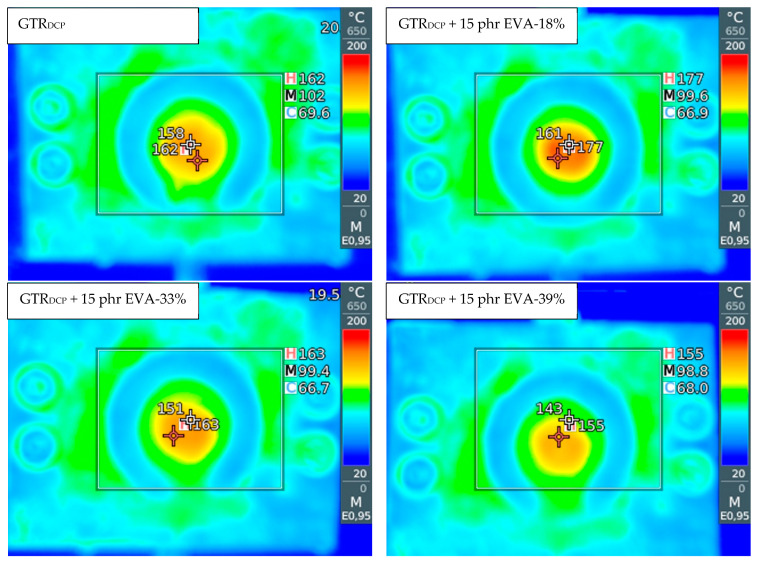
The infrared camera images for samples: GTR_DCP_; GTR_DCP_ + 15 phr EVA-18%; GTR_DCP_ + 15 phr EVA-33%; GTR_DCP_ + 15 phr EVA-39%.

In order to evaluate the change in temperature distribution in the modified GTR after auto-thermal extrusion, an infrared camera was used, and the obtained images are presented in Figure 1. It was observed that at a higher content of thermoplastic modifier, the EVA copolymer acted like binder or plasticizer, which affected the final temperature of the material formulated in the solid profiles. The temperature and specific mechanical energy consumption measured during the modification of GTR are summarized in Table 3. The measurement of specific mechanical energy (*SME*) provides information about extrusion process characteristics, which is very useful during up-scaling of research studies (estimation of production energetic costs). The *SME* parameter is correlated to the mechanical energy dissipated into the heat of the material inside the extruder barrel [40].

The temperature of the modified GTR after extrusion was in the range of 150–174 °C (160 °C for the GTR_DCP_ sample), which indicates that the temperature of the processed material increased for 20–44 °C compared to barrel temperature set on each section before process stabilization (60/85/105/115/110/115/130/130/120/120 °C). This also confirmed that the conducted process is auto-thermal, and heat is generated only by internal friction of the cross-linked GTR inside the extruder barrel.

The lowest temperature of the material after extrusion was measured for samples with 10 phr and 15 phr of EVA-39%, while the highest was for samples with 10 and 15 phr of EVA-18%. This is related to the differences in the melt flow index and melting temperature of the used EVA copolymers. EVA-18% is characterized by the lowest melt flow index and the highest melting temperature among used EVA copolymers (see Table 1), which might enhance the interfacial interactions enforced by shear force during extrusion of GTR with EVA-18%.

As presented in Table 2, for the studied materials, the SME parameter was in the range of 0.120–0.177 kWh/kg (0.131 kWh/kg for the GTR_DCP_ sample). It was observed that changes in the SME values corresponded to the material temperature measurements. The highest values of the SME parameter were determined for the samples characterized by the highest temperature after extrusion—samples modified with 10 and 15 phr of EVA-18%, while the lowest was for samples with 10 phr and 15 phr of EVA-39%.

### 3.2. Curing Characteristics of Modified GTR

**Figure 2:**
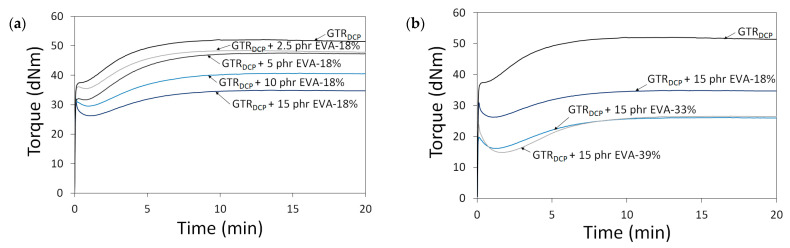
Curing curves for modified GTR determined at 170 °C as a function of: EVA content (0–15 phr) (**a**) and EVA grade (VA content: 18–39%) (**b**).

In Figure 2, the curing curves of the modified GTR were a function of A—EVA content (0–15 phr) and B—EVA grade (VA content: 18–39%). All processed materials showed an increase in torque as a function of time related to their cross-linking inside the rheometer chamber. This confirms that the reaction of DCP with the GTR/EVA system was limited during sample preparation by adiabatic extrusion. It was observed that the higher content of EVA-18% resulted in a proportional decrease in minimal torque (M_L_). The determined M_L_ parameter was 37.8 dNm for the GTR_DCP_ sample, 35.5 dNm for GTR_DCP_ + 2.5 phr EVA-18%, 31.7 dNm for GTR_DCP_ + 5 phr EVA-18%, 29.5 dNm for GTR_DCP_ + 10 phr EVA-18%, and 26.2 dNm for GTR_DCP_ + 15 phr EVA-18%, respectively. This tendency indicates better processing of the GTR modified by the EVA copolymer. As can be observed, increasing the content of vinyl acetate in the EVA copolymer from 18% to 39% shifts M_L_ toward lower values for the sample with 15 phr of EVA-33% (16.2 dNm), and the sample with 15 phr of EVA-39% (14.8 dNm). As presented in Table 1, the increasing content of VA in the EVA copolymer affects its higher melt flow index (in the range: 1.7–60 g/10 min) and lower melting point (from 87 to 48 °C), which improves the processing of the modified GTR. Moreover, the trend of the curing curves showed that higher content of EVA and also the increasing content of vinyl acetate in the EVA copolymer resulted in a decrease in maximal torque (M_H_) and torque increment (ΔM=M_H_-M_L_). However, in the studied conditions, the differences between EVA-33% and EVA-39% were negligible. Regardless of EVA copolymer grade, the scorch time and optimal cure time increased with a higher content of thermoplastic modifier. For example, the scorch time and optimal cure time for the GTR_DCP_ + 2.5 phr EVA-18% sample were 1.7 and 6.6 min, while for the GTR_DCP_ + 15 phr EVA-18% sample, the values of these parameters were 2.6 and 8.2 min, respectively.

For all the studied systems, the scorch time varied in the range of 1.4 min (for the GTR_DCP_ sample) to 3.2 min (for the GTR_DCP_ + 15 phr EVA-39% sample), while the optimal cure time was in the range of 6.5 min (for the GTR_DCP_ sample) to 9.1 min (for the GTR_DCP_ + 15 phr EVA-39% sample). This tendency can be related to co-cross-linking between the GTR and EVA phases in the presence of DCP, characterized by different reaction kinetics. Similar observations were described by Bianchi et al. [41], who studied the cross-linking kinetics of EVA/GTR blends (GTR content from 0 up to 75 wt.%) using an oscillating disc rheometer and differential scanning calorimetry.

### 3.3. Physico-Mechanical Properties of Modified GTR

**Figure 3:**
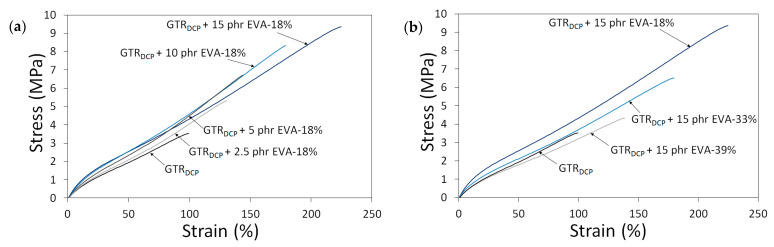
Stress–strain curves as a function of: EVA content (0–15 phr) (**a**) and EVA grade (VA content: 18–39%) (**b**).

To highlight the differences in the tensile properties of modified GTR as a function of EVA content and EVA grade, stress–strain curves are presented in Figure 3. The trends of the stress–strain curves show that the tensile strength of modified GTR usually increases with a higher content of EVA. Moreover, the tensile parameters decrease for samples containing EVA copolymers with a higher content of vinyl-acetate, which can be related to the lower compatibility of non-polar GTR with EVA-39% characterized by higher polarity.

**Figure 4:**
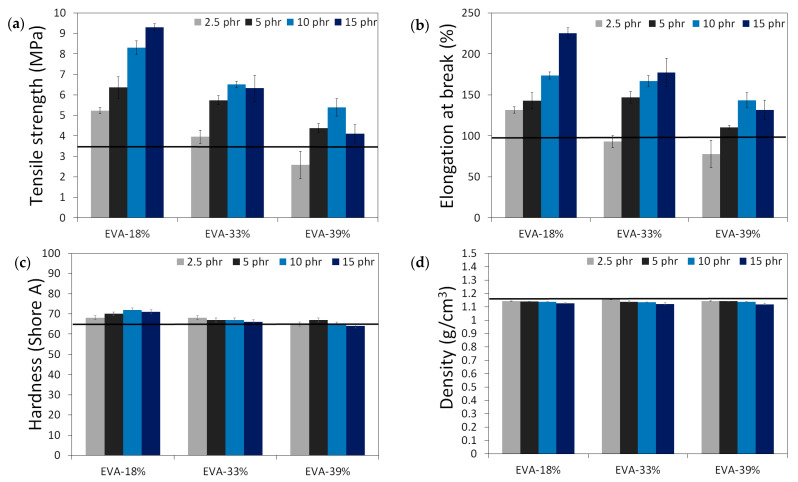

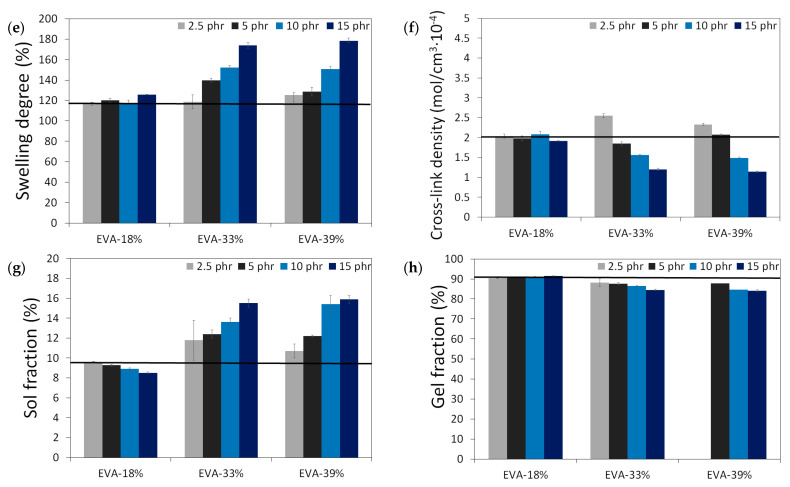
Physico-mechanical properties of modified GTR (black line represents sample with EVA): tensile strength (MPa) (**a**); elongation at break (%) (**b**); hardness (Shore A) (**c**); density (g/cm^3^) (**d**); swelling degree (%) (**e**), cross-link density (mol/cm^3^ 10^−4^) (**f**), sol fraction content (%) (**g**) and gel fraction content (%) (**h**).

The results of the physico-mechanical properties of modified GTR, including tensile strength (MPa), elongation at break (%), hardness (Shore A), density (g/cm^3^), swelling degree (%), cross-link density (mol/cm^3^ 10^−4^), and sol and gel fraction content (%) as a function of EVA content and EVA grade, are summarized in the graphs presented in Figure 4. The black line in the presented graphs represents the reference sample without an EVA copolymer modifier (GTR_DCP_ sample).

Modified GTR was characterized by tensile strength in the range of 2.6–9.3 MPa (3.5 MPa for the GTR_DCP_ sample); elongation at break in the range of 78–225% (98% for the GTR_DCP_ sample); hardness in the range of 64–72 Shore A (65 Shore A for the GTR_DCP_ sample); density in the range of 1.118–1.154 g/cm^3^ (1.154 g/cm^3^ for the GTR_DCP_ sample); swelling degree in the range of 117–178% (117% for the GTR_DCP_ sample); cross-link density in the range of 1.14–2.55 mol/cm^3^ 10^−4^ (2.01 mol/cm^3^ 10^−4^ for the GTR_DCP_ sample); sol fraction in the range of 8.5–15.9% (9.5% for the GTR_DCP_ sample), and gel fraction in the range of 84.1–91.5% (90.5% for the GTR_DCP_ sample). The tensile properties of the GTR modified EVA copolymers in most compositions an exception was the sample with 2.5 phr of EVA-39%) were higher compared to the reference sample (GTR_DCP_). Moreover, the prepared modified GTR showed tensile properties comparable or even higher than those of commercially available reclaimed rubbers (~4–5 MPa) [30], which fully justifies the application of GTR modification.

### 3.4. VOCs’ Emission Profiles Determined for Modified GTR

To determine the emission of volatile organic compounds emitted from modified GTR, three kinds of sampling protocols were used. During extrusion, we used a Radiello^®^ diffusing sampling system, while the emission of uncured and cured samples of modified GTR was evaluated using stationary microchambers and static headspace. The collected samples were analyzed by gas chromatography with a flame ionization detector (GC-FID), and gas chromatography with mass spectrometry (GC-MS). GC-FID analysis provided information about total volatile organic compounds (TVOCs) released from the uncured and cured modified reclaimed rubbers, while the GC-MS allowed for the investigation of the chemical structure of volatile organic compounds emitted from the studied materials during reactive extrusion.

**Figure 5:**
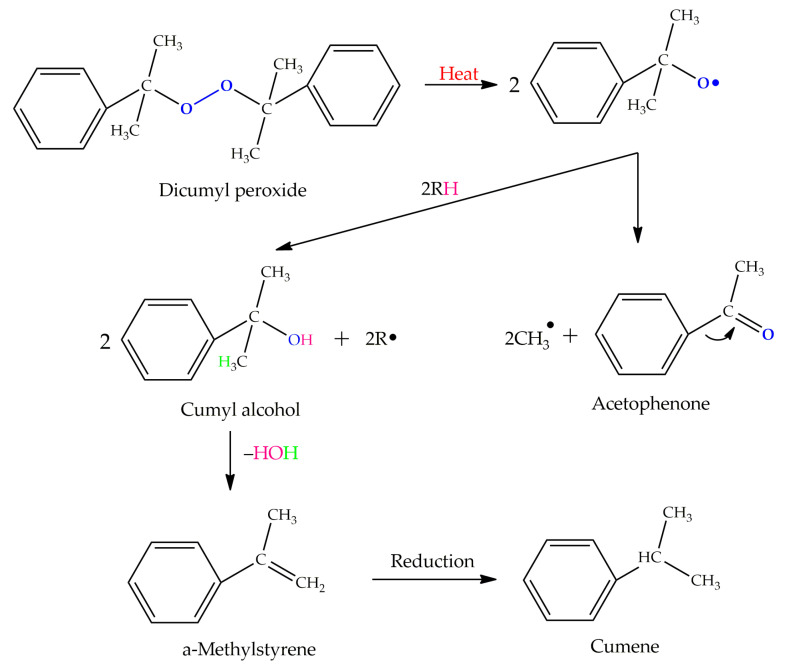
Mechanism of dicumyl peroxide decomposition.

Table 4 presents the chemical structures of the volatile organic compounds collected by Radiello^®^ and determined by GC-MS analysis. The identified compounds are mostly oxidative degradation products of GTR and DCP decomposition by-products. For GTR, they can be classified into three basic groups depending on their source: (i) natural rubber degradation products, which include: methyl isobutyl ketone, undecane, dodecane, tridecane, tetradecane; (ii) aromatic compounds that are decomposition products of styrene-butadiene rubber (benzene, toluene, styrene, xylene, hexylbenzene); and (iii) vulcanization accelerator residue, which is represented by benzothiazole. The presence of this type of substance indicates the occurrence of the reclaiming process, while the intensity of the detected accelerator residues can be an indicator of the degree of devulcanization [20,28]. Volatile organic compounds such as cumene, α-methylstyrene, acetophenone, and α-cumyl alcohol were identified as decomposition by-products of dicumyl peroxide [42,43]. The mechanism of dicumyl peroxide decomposition is shown in Figure 5. The intensity of the emitted DCP decomposition by-products was much higher in the cured materials, which might be due to the partial decomposition of DCP during extrusion of the studied materials and as a consequence of co-cross-linking between GTR and EVA copolymer.

Total volatile organic compounds and acetophenone concentration (a by-product of DCP decomposition) emitted from the modified reclaimed rubber are summarized in Table 5. It should be mentioned that both parameters were investigated by different methodologies. TVOCs were determined using an emission chamber-GC-FID system, while acetophenone concentration was studied by HS-GC-MS.

The TVOC parameter was in the range of 15.0 to 64.6 μg/g. It was estimated that the TVOCs released from the cured materials were on average two times higher as compared to those of uncured modified GTR. Moreover, as could be expected, the concentration of acetophenone in the cured samples was much higher than that of uncured samples. This phenomenon is related to the higher pressing temperature, which contributes to the increased rate of cross-linking. Additionally, some peroxide-induced degradation might simultaneously occur in the studied materials during their cross-linking. Moreover, a part of the volatile organic compounds was released into the atmosphere during processing and curing. The abovementioned factors might have caused there to be no simple correlation between the composition of modified GTR (EVA copolymer content or grade) and the concentration of volatile organic compounds.

### 3.5. Storage Stability Assessment of Modified GTR

Modified GTR was stored in closed 10L metal containers for 3 months at ambient temperature. For sample preparation, the modified GTR was collected after 1, 2, and 3 months, respectively.

Table 6 summarizes the curing parameters determined shortly after extrusion and after 1, 2, and 3 months. It can be seen from the averaged values that the differences of all parameters measured at different time intervals are insignificant. It is worth mentioning that the measurement was carried out once for each sample, so the observed deviations are fully justified. However, as can be observed for the GTR_DCP_ sample, the results showed the highest variations, especially regarding the minimal torque and torque increment parameters. This can be due to two main factors. The first is the complex composition of the GTR received as a mixture of passenger car tires and truck tires. The second is the form of the studied materials, especially their cohesion and interfacial adhesion, which increased with a higher content of EVA. The studied materials have a form of ribbon; however, for GTR_DCP_, there was a tendency to separation and agglomerate formulation, which might have an impact on torque values measured by oscillating disc rheometry measurements (the scorch time and optimal cure time parameters were very similar). One solution to this problem is the application of moving die rheometry or a rubber process analyzer, in which the construction of chambers allows measurements of materials with limited flowability.

It was observed that the higher content of EVA copolymers as thermoplastic modifiers resulted in more consistent materials, characterized by enhanced processing (defined as minimal torque).

To summarize this section, the obtained results allow for the conclusion that the storage time did not affect the curing characteristics of the materials modified by EVA, which indicates that the application of a thermoplastic modifier enhances and stabilizes the processing and curing behavior during storage.

The mechanical properties (tensile strength, elongation at break, and hardness) as a function of storage time are shown in Table 7. The determined values of tensile strength, elongation at break, and hardness differ slightly within a given sample. As in the case of curing parameters, these differences are slight and random (they do not follow any trend). This is due to the fact that the basic raw material (GTR) is hardly reproducible. However, the essential result is the preservation of a constant tendency in the properties of the samples determined by the different content and type of copolymer EVA.

Similar observations were described by Lu et al. [48], who proved that the tensile strength and elongation at break of reclaimed rubber remained unchanged during up to 60 days of storage. Moreover, the authors observed that the sol fraction content was reduced and the cross-linking density increased during storage. The authors assumed it was due to slow recombination and aggregation of the molecular fragments with free radicals occurring in the reclaimed rubber.

### 3.6. Characterization of SBR/Modified GTR and NR/Modified GTR Blends

Taking into account the performance properties and considering the potential costs of modified GTR production, a sample coded as GTR_DCP_ + 2.5 phr EVA-18% was selected for further applications in rubber compounds. The processing and physico-mechanical properties of blends based on styrene-butadiene rubber (SBR) and natural rubber (NR) with modified GTR in ratios of 75/25, 50/50, and 25/75 wt.% were investigated, and the obtained results are summarized in Table 8. SBR and NR were chosen for the blend formulation due to their common application in the tire industry. It should be mentioned that the studied rubber/modified GTR blends were prepared without using any additional curing system in order to verify the possibility of curing the studied blends by dicumyl peroxide present in modified GTR.

It was found that the application of modified GTR has a significant impact on differences in the processing of the studied SBR/modified GTR blends and NR/modified GTR blends. A higher content of modified GTR in the SBR/modified GTR blends resulted in a proportional increase in Mooney viscosity (55.6–124.7 MU) and minimal torque (9.2–19.9 dNm). For the NR/modified GTR blends, the effect of modified GTR content on the values of these processing parameters was negligible. NR/modified GTR was characterized by Mooney viscosity in the range of 21.2–27.6 MU and minimal torque in the range of 4.7–6.2 dNm.

Regardless of the rubber matrix used (SBR or NR), for curing kinetics, the same trends were observed. A higher content of modified GTR resulted in increasing values of maximal torque (for SBR/modified GTR: 22.4–45.3 dNm and for NR/modified GTR: 12.5–34.4 dNm), torque increment (for SBR/modified GTR: 13.2–25.4 dNm and for NR/modified GTR: 7.8–28.2 dNm), and cure rate index (for SBR/modified GTR: 27.1–35.0 min^−1^ and for NR/modified GTR: 25.4–35.0 min^−1^), which is due to the higher cross-linking of the studied materials. At the same time, samples with a higher content of modified GTR scorch time (in the range of 0.7–1.5 min) and optimal cure time (in the range o: 3.6–5.4 min) values were reduced. Moreover, it was found that a higher content of modified GTR increases the thermal aging resistance factor, from 0.0% for elastomer/modified GTR in the ratio 75/25 wt.% to 1.3% for blends in the ratio 25/75 wt.% This phenomenon is related to a higher concentration of DCP in the elastomer/modified GTR blends, which changes proportionally with the content of modified GTR.

The results of the physico-mechanical properties measurements showed that, regardless of the rubber matrix used (SBR or NR), a higher amount of modified GTR in the elastomer/modified GTR blends resulted in an increase in tensile strength, modulus at 100%, modulus at 300%, hardness, density, and abrasion resistance. Simultaneously, an opposite trend was observed in the case of such parameters as elongation at break, swelling degree, and sol fraction (for NR/modified GTR blends, the sol fraction parameter was independent of ratio). It can be concluded that the obtained results are mainly related to the cross-linking efficiency of the studied systems. The tensile strength (in the range of 1.7–6.4 MPa) of the prepared rubber/modified GTR values are similar to those described in the literature. Sreeja and Kutty [49] investigated SBR/reclaimed rubber blends, and their results showed that tensile strength for such a system was 1.9 MPa for the sample without reclaimed rubber and 5.1 MPa for the sample with 80 phr of reclaimed rubber. The same authors studied NR/reclaimed rubber blends [50] and found that a higher content of reclaimed rubber in NR resulted in the deterioration of mechanical properties from ~14.8 MPa for the sample with 20 phr of reclaimed rubber to ~12.5 MPa for the sample with 80 phr of reclaimed rubber (the estimated values were read from the figure). Similar observations were described in a recent work by Zhao et al. [51] who studied NR/reclaimed rubber and NR/ground tire rubber blends. The authors demonstrated that the tensile strength parameter for the NR/reclaimed rubber blend in a ratio of 90/10 was ~10.2 MPa, while for the same composition in a ratio of 10/90 it was ~8.1 MPa (the estimated values were read from the figure). Higher deterioration of tensile properties was observed in the case of NR/ground tire rubber blends, for which ~5.0 MPa and ~1.2 MPa (the estimated values were read from the figure) were measured for composition in ratios of 90/10 and 10/90, respectively.

**Figure 6:**
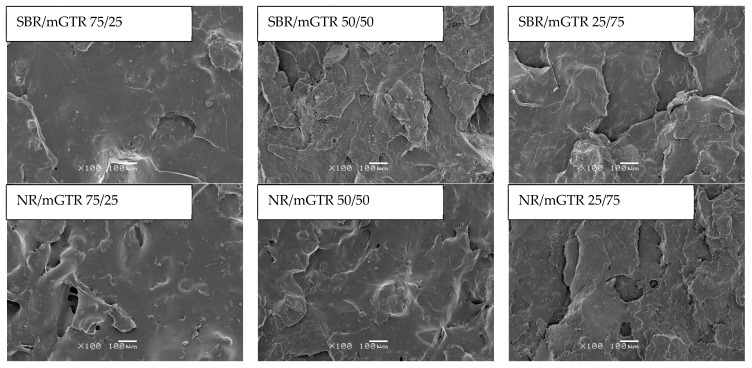
SEM images of rubber/modified GTR blend surface areas after tensile tests (magnification x100).

For a better understanding of the breaking mechanism of the studied materials during the tensile test (cross-head speed: 500 mm/min), the scanning electron microscopy images of surface area perpendicular to the direction of strain are presented in Figure 6. As can be observed, regardless of the rubber matrix used (SBR or NR), the increasing content of modified GTR in the rubber/modified GTR blends resulted in a more developed surface and cohesion crack, which indicated partial co-cross-linking between the rubber matrix and modified GTR. The efficacy of this process increases with the modified GTR content a (higher DCP amount in the system). However, it is worth mentioning that for the SBR/modified GTR blend, the surface seems to be more developed compared to the NR/modified GTR blends, which can be related to significant differences in the processing properties of the studied blends. As previously mentioned, the NR/modified GTR blends showed a lower Mooney viscosity (in the range of 21.2–27.6 MU) and minimal torque (in the range of 4.5–6.2 dNm) in comparison to the SBR/modified GTR blends (Mooney viscosity: 55.6–127.3 MU and minimal torque: 9.2–19.9 dNm). This feature has a significant impact on the dispersion level of GTR in the rubber matrix, encapsulation of GTR by the rubber matrix, and co-cross-linking efficiency between rubber and modified GTR. Consequently, the combination of the abovementioned factors affects the morphology and the final performance properties of the prepared materials.

## 4. Conclusions

In this work, the efficiency of GTR modification in the presence of dicumyl peroxide and EVA copolymer was analyzed as a function of EVA content (0–15 phr) and EVA grade (VA content: 18–39%). Modification of GTR was performed by auto-thermal extrusion at a throughput rate of 5.0–5.75 kg/h. In the studied conditions, the internal friction of the cross-linked GTR particles inside the extruder barrel resulted in an increase in modified GTR above the setpoint (temperature set on the die: 120 °C, the temperature of mGTR after extrusion: 150–174 °C). It has been shown that the EVA copolymer content and structure have a significant impact on processing, curing behavior, and the physical–mechanical properties of modified GTR. The results indicated that GTR modifications using a higher content of EVA with a lower VA content (highest tensile strength and elongation at break) are the most effective.

Among the studied materials, the best mechanical properties were seen in the sample modified with 15 phr of EVA-18% copolymer, which was characterized by a tensile strength of 9.3 MPa and elongation at break of 225%.

The proposed method allows the manufacturing of materials that are stable during storage (for at least three months) with performance properties competitive, or even superior, to commercially available reclaimed rubbers. Moreover, it was found that the obtained modified GTR can be easily mixed with a fresh rubber matrix (SBR, NR), which resulted in co-cross-linking between the two phases without the need for modification of the entire system by an additional curing system.

The results obtained indicate that the modification, by continuous methods, of GTR with thermoplastics has the potential for efficient rubber waste management. It seems that future research in this field should be focused on: (i) suitable selection and optimization of additives (e.g., thermoplastics, curing agents, plasticizers, etc.) used during GTR modification; (ii) investigation of the impact of rubber compound composition (e.g., filler, curing additives, etc.) on rubber matrix-modified GTR interfacial interactions, (iii) up-scaling of the obtained research and its industrial implementation, and (iv) searching for special applications of modified GTR allowing the further development of waste rubber upcycling technologies (e.g., materials dedicated to 3D printing [52], or self-healing [53] and thermo-responsive shape-memory materials [54]).

## Figures and Tables

**Table 1:** Properties of the used EVA copolymers.

Properties	Standard	Additive		
		FL00218 *	UL04533EH2 *	UL05540EH2 *
Abbreviation in this study	-	EVA-18%	EVA-33%	EVA-39%
Vinyl acetate content (wt.%)	Producer method	18	33	39
Density at 25 °C (g/cm^3^)	ASTM D1505	0.940	0.956	0.966
MFI_190 °C, 2.16 kg_ (g/10 min)	ASTM D1238	1.7	45	60
Vicat softening temperature (°C)	ASTM D1525	62	33	-
Melting temperature (°C)	Producer method	87	61	48

* Information from technical data sheets provided by producer ExxonMobil Chemical.

**Table 2:** Sample preparation protocol and methodology used for modified GTR and rubber/modified GTR blends.

Methodology	Standard	Modified GTR	Rubber/Modified GTR Blends
**Sample preparation**	-	Twin-screw extruder + compression molding	Two-roll mills + compression molding
**Processing assessment**			
Energy consumption + IR camera	-	X	-
Mooney viscosity	ISO 287	-	X
Curing characteristics	ISO 3417	X	X
**Physico-mechanical properties**			
Density	ISO 2781	X	X
Swelling behavior	-	X	X
Tensile tests	ISO 37	X	X
Hardness	7619-1	X	X
Abrasion resistance	ISO 4649	-	X
**Additional methods**			
VOCs emission characteristics by GC-MS and GC-FID	-	X	-
Microstructure evaluation by SEM	-	-	X

**Table 3:** Temperature and specific mechanical energy consumption measured during modification of GTR.

Sample Code	EVA Grade	EVA Content (phr)	Temperature at Die (°C)	*SME* (kWh/kg)
GTR_DCP_	-	0	160 ± 1	0.131 ± 0.003
GTR_DCP_ + 2.5 phr EVA-18%	EVA-18%	2.5	164 ± 6	0.125 ± 0.003
GTR_DCP_ + 5 phr EVA-18%		5	172 ± 3	0.171 ± 0.010
GTR_DCP_ + 10 phr EVA-18%		10	174 ± 1	0.177 ± 0.002
GTR_DCP_ + 15 phr EVA-18%		15	173 ± 2	0.171 ± 0.003
GTR_DCP_ + 2.5 phr EVA-33%	EVA-33%	2.5	165 ± 4	0.160 ± 0.012
GTR_DCP_ + 5 phr EVA-33%		5	172 ± 5	0.168 ± 0.004
GTR_DCP_ + 10 phr EVA-33%		10	164 ± 3	0.154 ± 0.001
GTR_DCP_ + 15 phr EVA-33%		15	158 ± 2	0.146 ± 0.003
GTR_DCP_ + 2.5 phr EVA-39%	EVA-39%	2.5	167 ± 4	0.147 ± 0.007
GTR_DCP_ + 5 phr EVA-39%		5	161 ± 4	0.149 ± 0.004
GTR_DCP_ + 10 phr EVA-39%		10	150 ± 2	0.129 ± 0.006
GTR_DCP_ + 15 phr EVA-39%		15	152 ± 3	0.120 ± 0.005

**Table 4:** Volatile organic compounds identified by GC-MS measurement during preparation (reactive extrusion) of modified GTR—sampling via Radiello^®^ diffusing sampling system.

Retention Time (min)	Identified Compound	Chemical Structure	Molecular Weight (g/mol)	Match Quality (%)	Source	References
4.34	Benzene	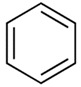	78.11	94	styrene-butadiene rubber present in GTR	[20]
5.38	Methyl Isobutyl Ketone	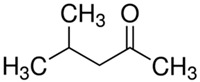	100.16	90	natural rubber and anti-aging agents present in GTR	[44,45]
6.02	Toluene	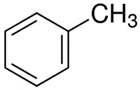	92.14	95	styrene-butadiene rubber present in GTR	[20,46]
7.87	Xylene	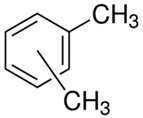	106.17	97	styrene-butadiene rubber present in GTR	[20,46]
8.20	Styrene	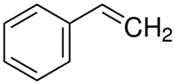	104.15	97	styrene-butadiene rubber present in GTR	[20,46]
8.94	Cumene	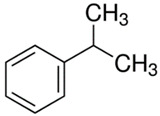	120.19	91	dicumyl peroxide decomposition	[41]
10.29	α-Methylstyrene	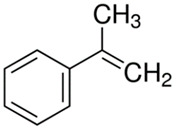	118.18	93	dicumyl peroxide decomposition	[47]
11.78	Acetophenone	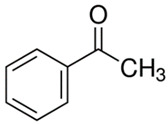	120.15	97	dicumyl peroxide decomposition	[47]
12.16	α-Cumyl alcohol	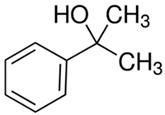	136.91	91	dicumyl peroxide decomposition	[47]
12.74	Undecane	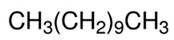	156.31	94	EVA copolymer andnatural rubber present in GTR	-
14.25	Dodecane	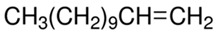	168.32	95	EVA copolymer andnatural rubber present in GTR	-
14.41	Benzothiazole	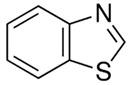	135.19	94	vulcanization accelerators present in GTR	[5,20,44]
14.98	Hexylbenzene	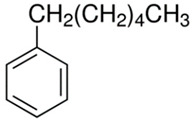	162.27	95	styrene-butadiene rubber present in GTR	-
15.49	Tridecane	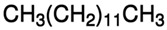	184.36	96	EVA copolymer andnatural rubber present in GTR	-
16.57	Tetradecane	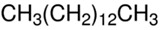	198.39	98	EVA copolymer andnatural rubber present in GTR	-

**Table 5:** TVOCs and acetophenone level determined for uncured and cured modified GTR.

Sample Code	EVA Grade	EVA Content (phr)	TVOCs (μg/g)		Acetophenone (mg/kg)	
			Uncured	Cured	Uncured	Cured
GTR_DCP_	-	0	15.0	30.6	4.3	112.6
GTR_DCP_ + 2.5 phr EVA-18%	EVA-18%	2.5	23.3	48.9	3.9	23.4
GTR_DCP_ + 5 phr EVA-18%		5	18.4	60.4	11.0	162.2
GTR_DCP_ + 10 phr EVA-18%		10	25.3	39.2	3.6	38.3
GTR_DCP_ + 15 phr EVA-18%		15	23.7	44.5	-	77.5
GTR_DCP_ + 2.5 phr EVA-33%	EVA-33%	2.5	29.2	49.6	6.8	40.1
GTR_DCP_ + 5 phr EVA-33%		5	22.2	53.6	7.0	22.4
GTR_DCP_ + 10 phr EVA-33%		10	22.9	50.5	12.8	68.3
GTR_DCP_ + 15 phr EVA-33%		15	22.6	37.9	12.1	23.1
GTR_DCP_ + 2.5 phr EVA-39%	EVA-39%	2.5	25.0	64.6	3.9	64.6
GTR_DCP_ + 5 phr EVA-39%		5	24.2	58.0	3.3	41.1
GTR_DCP_ + 10 phr EVA-39%		10	17.8	63.3	3.6	111.0
GTR_DCP_ + 15 phr EVA-39%		15	18.4	64.0	5.3	40.0
**Technique**			emission chamber-GC-FID		HS-GC-MS	
**Conditioning temperature**			40 °C		100 °C	
**Conditioning time**			20 min		20 min	
**Carrier gas flow rate**			25 mL/min		2 mL/min	
**Oven temperature** **program**			40 °C for 1 min 10 °C/min up to 280 °C 20 °C/min up to 300 °C for 3 min		40 °C for 4.5 min 10 °C/min up to 200 °C 30 °C/min up to 290 °C for 5 min	

**Table 6:** Curing characteristics of modified GTR as a function of storage time.

Sample Code	Curing Parameters	Curing Characteristics as a Function of Storage Time				Average
		Few Days	1 Month	2 Months	3 Months	
GTR_DCP_	M_L_ (dNm)	37.8	38.5	15.4	24.8	29.1 ± 11.1
	ΔM (M_H_-M_L_) (dNm)	14.2	13.3	6.1	9.8	10.9 ± 3.7
	Scorch time (min)	1.4	1.5	1.9	2.0	1.7 ± 0.3
	Optimal cure time (min)	6.5	6.7	6.1	6.4	6.4 ± 0.3
GTR_DCP_ + 2.5 phr EVA-18%	M_L_ (dNm)	35.5	37.9	35.3	35.6	36.1 ± 1.2
	ΔM (M_H_-M_L_) (dNm)	12.9	12.0	9.9	11.3	11.5 ± 1.3
	Scorch time (min)	1.7	1.9	2.1	2.2	2.0 ± 0.2
	Optimal cure time (min)	6.6	6.6	7.0	7.5	6.9 ± 0.4
GTR_DCP_ + 5 phr EVA-18%	M_L_ (dNm)	31.7	35.3	31.6	34.2	33.2 ± 1.8
	ΔM (M_H_-M_L_) (dNm)	15.8	12.8	9.8	9.8	12.1 ± 2.9
	Scorch time (min)	1.7	2.0	2.2	2.5	2.1 ± 0.3
	Optimal cure time (min)	7.4	7.1	7.3	7.9	7.4 ± 0.3
GTR_DCP_ + 10 phr EVA-18%	M_L_ (dNm)	29.5	35.2	29.8	32.2	31.7 ± 2.6
	ΔM (M_H_-M_L_) (dNm)	11.2	10.3	7.7	7.4	9.2 ± 1.9
	Scorch time (min)	2.3	2.5	2.6	2.8	2.6 ± 0.2
	Optimal cure time (min)	8.4	7.4	7.6	7.9	7.8 ± 0.4
GTR_DCP_ + 15 phr EVA-18%	M_L_ (dNm)	26.2	30.7	28.2	29.7	28.7 ± 2.0
	ΔM (M_H_-M_L_) (dNm)	8.7	6.4	5.6	5.4	6.5 ± 1.5
	Scorch time (min)	2.6	3.0	3.4	3.7	3.2 ± 0.5
	Optimal cure time (min)	8.2	7.7	7.9	8.4	8.1 ± 0.3
GTR_DCP_ + 2.5 phr EVA-33%	M_L_ (dNm)	29.8	33.0	31.0	31.5	31.3 ± 1.3
	ΔM (M_H_-M_L_) (dNm)	14.0	11.0	8.5	9.7	10.8 ± 2.4
	Scorch time (min)	1.8	2.3	2.6	2.6	2.3 ± 0.4
	Optimal cure time (min)	7.0	7.3	7.5	7.4	7.3 ± 0.2
GTR_DCP_ + 5 phr EVA-33%	M_L_ (dNm)	27.2	28.1	28.2	28.0	27.9 ± 0.5
	ΔM (M_H_-M_L_) (dNm)	8.7	9.0	8.5	8.0	8.6 ± 0.4
	Scorch time (min)	2.4	2.7	3.0	3.0	2.8 ± 0.3
	Optimal cure time (min)	7.9	7.6	8.4	8.0	8.0 ± 0.3
GTR_DCP_ + 10 phr EVA-33%	M_L_ (dNm)	21.0	22.7	22.6	22.4	22.2 ± 0.8
	ΔM (M_H_-M_L_) (dNm)	11.1	9.4	9.5	10.2	10.1 ± 0.8
	Scorch time (min)	2.5	3.0	3.0	2.9	2.9 ± 0.2
	Optimal cure time (min)	8.4	8.4	8.8	8.3	8.5 ± 0.2
GTR_DCP_ + 15 phr EVA-33%	M_L_ (dNm)	16.1	17.9	17.2	17.1	17.1 ± 0.7
	ΔM (M_H_-M_L_) (dNm)	9.9	10.3	9.0	9.9	9.8 ± 0.6
	Scorch time (min)	2.8	2.9	3.3	2.9	3.0 ± 0.2
	Optimal cure time (min)	8.5	8.7	9.1	8.7	8.8 ± 0.3
GTR_DCP_ + 2.5 phr EVA-39%	M_L_ (dNm)	33.6	32.2	35.0	32.9	33.4 ± 1.2
	ΔM (M_H_-M_L_) (dNm)	10.9	10.5	10.9	12.2	11.1 ± 0.7
	Scorch time (min)	2.2	2.2	2.1	1.8	2.1 ± 0.2
	Optimal cure time (min)	7.0	7.0	7.6	7.3	7.2 ± 0.3
GTR_DCP_ + 5 phr EVA-39%	M_L_ (dNm)	27.7	26.0	30.3	29.0	28.3 ± 1.8
	ΔM (M_H_-M_L_) (dNm)	10.9	11.8	11.6	11.1	11.4 ± 0.4
	Scorch time (min)	2.6	2.5	2.5	2.1	2.4 ± 0.2
	Optimal cure time (min)	8.0	8.0	8.8	7.6	8.1 ± 0.5
GTR_DCP_ + 10 phr EVA-39%	M_L_ (dNm)	19.7	20.4	21.8	20.2	20.5 ± 0.9
	ΔM (M_H_-M_L_) (dNm)	12.0	12.8	12.2	12.5	12.4 ± 0.4
	Scorch time (min)	2.9	2.6	2.8	2.6	2.7 ± 0.2
	Optimal cure time (min)	8.6	8.9	8.9	8.8	8.8 ± 0.1
GTR_DCP_ + 15 phr EVA-39%	M_L_ (dNm)	14.8	15.5	15.7	14.7	15.2 ± 0.5
	ΔM (M_H_-M_L_) (dNm)	11.7	11.8	11.6	11.7	11.7 ± 0.1
	Scorch time (min)	3.2	3.1	3.2	3.0	3.1 ± 0.1
	Optimal cure time (min)	9.1	8.9	9.5	9.4	9.2 ± 0.3

**Table 7:** Performance properties of modified GTR as a function of storage time.

Sample Code	Performance Properties	Performance Properties as a Function of Storage Time				Average
		Few Days	1 Month	2 Months	3 Months	
GTR_DCP_	Tensile strength (MPa)	3.5 ± 0.2	3.6 ± 0.3	3.4 ± 0.1	3.0 ± 0.1	3.4 ± 0.3
	Elongation at break (%)	98 ± 3	92 ± 3	93 ± 5	90 ± 3	93 ± 3
	Hardness (Shore A)	65 ± 1	68 ± 1	67 ± 1	66 ± 1	67 ± 1
GTR_DCP_ + 2.5 phr EVA-18%	Tensile strength (MPa)	5.2 ± 0.2	5.1 ± 0.3	5.1 ± 0.2	4.9 ± 0.1	5.1 ± 0.1
	Elongation at break (%)	132 ± 4	114 ± 7	120 ± 3	134 ± 3	125 ± 10
	Hardness (Shore A)	68 ± 1	70 ± 1	70 ± 1	68 ± 1	69 ± 1
GTR_DCP_ + 5 phr EVA-18%	Tensile strength (MPa)	6.4 ± 0.5	6.3 ± 0.3	6.5 ± 0.5	6.3 ± 0.2	6.4 ± 0.1
	Elongation at break (%)	143 ± 10	138 ± 7	157 ± 8	151 ± 6	147 ± 8
	Hardness (Shore A)	70 ± 1	71 ± 1	71 ± 1	70 ± 1	71 ± 1
GTR_DCP_ + 10 phr EVA-18%	Tensile strength (MPa)	8.3 ± 0.3	8.0 ± 0.4	7.5 ± 0.2	8.2 ± 0.2	8.0 ± 0.4
	Elongation at break (%)	174 ± 4	171 ± 10	168 ± 9	194 ± 9	177 ± 12
	Hardness (Shore A)	72 ± 1	73 ± 1	72 ± 1	72 ± 1	72 ± 1
GTR_DCP_ + 15 phr EVA-18%	Tensile strength (MPa)	9.3 ± 0.2	8.7 ± 0.4	8.6 ± 0.2	8.3 ± 0.4	8.7 ± 0.4
	Elongation at break (%)	225 ± 7	220 ± 10	217 ± 4	224 ± 10	222 ± 4
	Hardness (Shore A)	71 ± 1	73 ± 1	72 ± 1	71 ± 1	72 ± 1
GTR_DCP_ + 2.5 phr EVA-33%	Tensile strength (MPa)	4.0 ± 0.3	3.9 ± 0.3	3.6 ± 0.4	4.1 ± 0.1	3.9 ± 0.2
	Elongation at break (%)	93 ± 7	103 ± 7	93 ± 7	103 ± 2	98 ± 6
	Hardness (Shore A)	68 ± 1	68 ± 1	69 ± 1	68 ± 1	68 ± 1
GTR_DCP_ + 5 phr EVA-33%	Tensile strength (MPa)	5.7 ± 0.2	5.0 ± 0.2	4.1 ± 0.2	4.3 ± 0.6	4.8 ± 0.7
	Elongation at break (%)	147 ± 7	135 ± 5	112 ± 6	124 ± 15	130 ± 15
	Hardness (Shore A)	67 ± 1	67 ± 1	67 ± 1	65 ± 1	67 ± 1
GTR_DCP_ + 10 phr EVA-33%	Tensile strength (MPa)	6.5 ± 0.1	7.1 ± 0.2	6.4 ± 0.2	6.8 ± 0.3	6.7 ± 0.3
	Elongation at break (%)	167 ± 7	187 ± 5	174 ± 5	178 ± 9	177 ± 8
	Hardness (Shore A)	67 ± 1	68 ± 1	67 ± 1	67 ± 1	67 ± 1
GTR_DCP_ + 15 phr EVA-33%	Tensile strength (MPa)	6.3 ± 0.6	6.7 ± 0.5	6.3 ± 0.3	7.0 ± 0.1	6.6 ± 0.3
	Elongation at break (%)	177 ± 17	185 ± 11	183 ± 6	203 ± 2	187 ± 11
	Hardness (Shore A)	66 ± 2	68 ± 1	68 ± 1	67 ± 1	67 ± 1
GTR_DCP_ + 2.5 phr EVA-39%	Tensile strength (MPa)	2.6 ± 0.7	4.1 ± 0.2	4.0 ± 0.3	2.6 ± 0.2	3.3 ± 0.8
	Elongation at break (%)	78 ± 17	96 ± 4	99 ± 5	79 ± 7	88 ± 11
	Hardness (Shore A)	65 ± 1	69 ± 1	68 ± 1	66 ± 1	67 ± 2
GTR_DCP_ + 5 phr EVA-39%	Tensile strength (MPa)	4.4 ± 0.2	4.8 ± 0.4	3.5 ± 1.0	4.2 ± 0.3	4.2 ± 0.5
	Elongation at break (%)	111 ± 2	120 ± 11	95 ± 23	115 ± 8	110 ± 11
	Hardness (Shore A)	67 ± 1	68 ± 1	68 ± 1	67 ± 1	68 ± 1
GTR_DCP_ + 10 phr EVA-39%	Tensile strength (MPa)	5.4 ± 0.4	5.8 ± 0.4	4.7 ± 0.8	5.2 ± 0.4	5.3 ± 0.5
	Elongation at break (%)	143 ± 9	147 ± 11	131 ± 15	148 ± 15	142 ± 8
	Hardness (Shore A)	65 ± 1	66 ± 1	67 ± 1	66 ± 1	66 ± 1
GTR_DCP_ + 15 phr EVA-39%	Tensile strength (MPa)	4.1 ± 0.4	5.6 ± 0.7	4.9 ± 0.7	4.9 ± 0.4	4.9 ± 0.6
	Elongation at break (%)	132 ± 12	171 ± 15	160 ± 19	163 ± 15	157 ± 17
	Hardness (Shore A)	64 ± 1	65 ± 1	64 ± 1	63 ± 1	64 ± 1

**Table 8:** Processing and physico-mechanical properties of rubber/modified GTR blends.

Property	SBR/Modified GTR Blends			NR/Modified GTR Blends		
	75/25	50/50	25/75	75/25	50/50	25/75
**Processing properties**						
Mooney viscosity ML(1+4) 100 °C (MU)	55.6 ± 0.6	81.8 ± 1.8	123.7 ± 2.0	26.4 ± 1.0	21.2 ± 1.0	27.6 ± 2.2
Minimal torque (dNm)	9.2	13.5	19.9	4.7	4.5	6.2
Maximal torque (dNm)	22.4	36.9	45.3	12.5	21.9	34.4
ΔM (M_H_-M_L_) (dNm)	13.2	23.4	25.4	7.8	17.4	28.2
Scorch time (min)	1.1	0.9	0.7	1.5	1.1	0.8
Optimal cure time (min)	4.8	3.9	3.6	5.4	4.4	3.7
Cure rate index (min^−1^)	27.1	32.4	35.0	25.4	30.7	35.0
Thermal aging resistance (%)	0.0	0.5	1.3	0.0	0.4	1.3
**Physico-mechanical properties**						
Tensile strength (MPa)	1.7 ± 0.1	3.1 ± 0.2	5.8 ± 0.1	2.0 ± 0.1	3.3 ± 0.6	6.4 ± 0.5
Modulus at 100% (MPa)	0.7 ± 0.1	1.3 ± 0.1	2.0 ± 0.1	0.4 ± 0.1	0.8 ± 0.1	1.8 ± 0.1
Modulus at 300% (MPa)	1.3 ± 0.1	-	-	0.9 ± 0.0	2.8 ± 0.2	-
Elongation at break (%)	451 ± 51	258 ± 15	274 ± 13	513 ± 9	333 ± 41	273 ± 11
Hardness (Shore A)	38 ± 2	49 ± 1	58 ± 1	25 ± 1	40 ± 1	55 ± 1
Density (g/cm^3^)	0.985 ± 0.002	1.038 ± 0.001	1.091 ± 0.002	0.958 ± 0.001	1.017 ± 0.004	1.081 ± 0.002
Abrasion resistance (mm^3^)	470 ± 66	292 ± 12	219 ± 3	- *	844 ± 46	377 ± 26
Swelling degree (%)	625 ± 5	293 ± 2	210 ± 2	637 ± 8	331 ± 4	193 ± 1
Sol fraction (%)	10.0 ± 0.2	7.3 ± 0.2	8.4 ± 0.1	7.6 ± 0.2	7.4 ± 0.0	8.1 ± 0.1

* Not measurable in the studied conditions.

## Data Availability

The data presented in this study are available on request from the corresponding author.
